# Focal Therapy for Prostate Cancer: Complications and Their Treatment

**DOI:** 10.3389/fsurg.2021.696242

**Published:** 2021-07-12

**Authors:** Arnas Rakauskas, Giancarlo Marra, Isabel Heidegger, Veeru Kasivisvanathan, Alexander Kretschmer, Fabio Zattoni, Felix Preisser, Derya Tilki, Igor Tsaur, Roderick van den Bergh, Claudia Kesch, Francesco Ceci, Christian Fankhauser, Giorgio Gandaglia, Massimo Valerio

**Affiliations:** ^1^Department of Urology, Lausanne University Hospital and University of Lausanne, Lausanne, Switzerland; ^2^Department of Urology, San Giovanni Battista Hospital, University of Turin, Turin, Italy; ^3^Department of Urology, Medical University Innsbruck, Innsbruck, Austria; ^4^Division of Surgery and Interventional Science, University College London, London, United Kingdom; ^5^Department of Urology, University College London Hospital, London, United Kingdom; ^6^Department of Urology, Ludwig-Maximilians-University of Munich, Munich, Germany; ^7^Urology Unit, Azienda Sanitaria Universitaria Integrata di Udine, Udine, Italy; ^8^Department of Urology, University Hospital Frankfurt, Frankfurt, Germany; ^9^Martini-Klinik Prostate Cancer Center, University Hospital Hamburg-Eppendorf, Hamburg, Germany; ^10^Department of Urology, University Hospital Hamburg-Eppendorf, Hamburg, Germany; ^11^Department of Urology and Pediatric Urology, Mainz University Medicine, Mainz, Germany; ^12^Department of Urology, Antonius Hospital, Utrecht, Netherlands; ^13^Department of Urology, University Hospital Essen, Essen, Germany; ^14^Division of Nuclear Medicine, IEO European Institute of Oncology Scientific Institute for Research, Hospitalization and Healthcare (IRCCS), Milan, Italy; ^15^Department of Urology, University Hospital Zürich, Zurich, Switzerland; ^16^Division of Oncology/Unit of Urology, Urological Research Institute, Scientific Institute for Research, Hospitalization and Healthcare (IRCCS) Ospedale San Raffaele, Milan, Italy

**Keywords:** prostate cancer, focal therapy, HIFU, cryotherapy, photodynamic therapy, complications

## Abstract

Focal therapy is a modern alternative to selectively treat a specific part of the prostate harboring clinically significant disease while preserving the rest of the gland. The aim of this therapeutic approach is to retain the oncological benefit of active treatment and to minimize the side-effects of common radical treatments. The oncological effectiveness of focal therapy is yet to be proven in long-term robust trials. In contrast, the toxicity profile is well-established in randomized controlled trials and multiple robust prospective cohort studies. This narrative review summarizes the relevant evidence on complications and their management after focal therapy. When compared to whole gland treatments, focal therapy provides a substantial benefit in terms of adverse events reduction and preservation of genito-urinary function. The most common complications occur in the peri-operative period. Urinary tract infection and acute urinary retention can occur in up to 17% of patients, while dysuria and haematuria are more common. Urinary incontinence following focal therapy is very rare (0–5%), and the vast majority of patients recover in few weeks. Erectile dysfunction can occur after focal therapy in 0–46%: the baseline function and the ablation template are the most important factors predicting post-operative erectile dysfunction. Focal therapy in the salvage setting after external beam radiotherapy has a significantly higher rate of complications. Up to one man in 10 will present a severe complication.

## Introduction

Prostate cancer is the second most commonly diagnosed cancer in men. Almost 1.3 million patients are diagnosed worldwide annually, and 360,000 deaths were related to prostate cancer in 2018 (3.8% of all deaths caused by cancer in men) (1). The prevalence of prostate cancer increases with age; screening is generally recommended in well-informed men with prolonged life expectancy. The incidence of prostate cancer diagnosis varies widely between different geographical areas, largely due to different habits in screening policies by mean of prostate-specific antigen (PSA) testing, and life expectancy (2). Decision making in men with localized disease is driven by risk classification, patient's comorbidities and preferences. At present, men with low-risk disease are usually offered active surveillance whereas men with intermediate to high-risk disease are offered radical treatment in the form of surgery or radiation therapy.

Radical prostatectomy and external beam radiotherapy (ERBT) are the two established treatment modalities for intermediate and high-risk localized prostate cancer. Both treatments lead to improved progression-free survival, but the competitive advantage against active surveillance is confined to men with aggressive features and/or very long life expectancy. On the other hand, the risk of genito-urinary toxicity, and rectal toxicity in case of ERBT, is substantial (3, 4). Consequently, tissue-preserving strategies have been developed to improve the therapeutic (risk to benefit) ratio of active treatment.

Focal therapy is an alternative strategy aiming to treat only the part of the prostate harboring clinically significant prostate cancer while preserving the rest of the gland. The objective is to retain the benefits of treating clinically significant cancer while minimizing the damage caused to the adjacent structures of the prostate by whole-gland treatments. Focal therapy, initially seen as an alternative to active surveillance, is now arguably seen as an alternative treatment modality for patients diagnosed with intermediate risk localized prostate cancer who would otherwise undergo radical therapy (5–8).

Recent advances in magnetic resonance imaging (MRI) and fusion targeted biopsy have allowed an accurate spatial localization of clinically significant lesions within the prostate (9–11). This revolution of the diagnostic paradigm shifting from a random to a targeted approach makes the case for an evolution in the therapeutic paradigm. Currently, each patient considered for focal therapy is required to undergo a rigorous diagnostic work-up. Prostate MRI followed by targeted and systematic prostate biopsy or mapping biopsy allow an accurate determination of the margins of the index lesion and rules out with high reliability non MRI visible clinically significant lesions (10, 11). The rationale of focal therapy is deemed reasonable by most; however, the protracted natural history observed in prostate cancer requires long-term evaluation in order to determine the oncological effectiveness of a novel treatment strategy.

Growing evidence in focal therapy has partly clarified its comparative effectiveness as compared to radical treatment options. Mid-term oncological effectiveness is promising; long-term outcomes are awaited. The largest systematic review reporting on different focal therapy outcomes in more than 2,000 patients was published by Valerio et al. (12). The biochemical recurrence ranged from 60 to 86% with the need for secondary focal or salvage treatments after primary treatment failure measured at 0–34%. The progression to metastatic disease was very low (0–0.3%) and cancer-specific survival was extremely high in this review. However, most of the studies had a retrospective design, a short follow-up time and a certain heterogeneity in defining outcome measures. A more recent systematic review including only comparative studies evaluating focal therapy against any standard treatment strategy has highlighted the lack of robust explanatory trials in the target population—men with clinically significant disease (13).

In contrast, the toxicity profile is well-established in randomized controlled trials and multiple prospective cohort studies employing validated patient-reported outcome measures (PROMs). The aim of this review was to summarize current available literature on complications following focal therapy.

## Methods

This narrative review is based on studies reporting on focal therapy short term and/or long-term functional outcomes (e.g., erectile dysfunction, incontinence) and/ or complications (infection, haematuria, bladder outlet obstruction, rectal toxicity etc.). The review was integrated by the experience of the authors in areas in which there is a lack of published evidence.

## Results

### Factors Influencing Toxicity

There are some factors influencing the toxicity after focal therapy. These include patient specific factors, cancer location, the amount of the tissue treated, and the source of the energy used.

Relevant patient related factors having an impact on postoperative toxicity are the size of the prostate, previous pelvic and prostate surgery, and predisposing conditions (pre-existing erectile dysfunction, lower tract urinary symptoms and neurological comorbidities) (8). The size of the prostate should always be precisely estimated prior to the treatment. Particularly large prostates might not be suitable for some energy sources or treatment templates; in such cases, patients are more at risk to develop significant lower urinary tract symptoms (LUTS) after treatment. Patient sexual and urinary functions should be well-documented with validated PROM prior to focal therapy. The most important determinant of erectile dysfunction after tissue preserving therapy is the preoperative erectile function status (14).

Cancer location is a key factor predicting the type and frequency of complications. Cancers located near the urethra, the bladder neck and the apical end are more difficult to be treated, and patients are more prone to develop postoperative irritative and obstructive LUTS. Cancers located close to the neurovascular bundles with capsule contact require extended ablation which may have an impact on erectile function recovery (15).

The amount of treated tissue has a significant impact on the toxicity: the more prostatic tissue is treated the more likely is to have postoperative complications. This has been clearly observed in studies comparing whole-gland to focal therapy strategies using the same treatment modalities (16).

**Figure 1:**
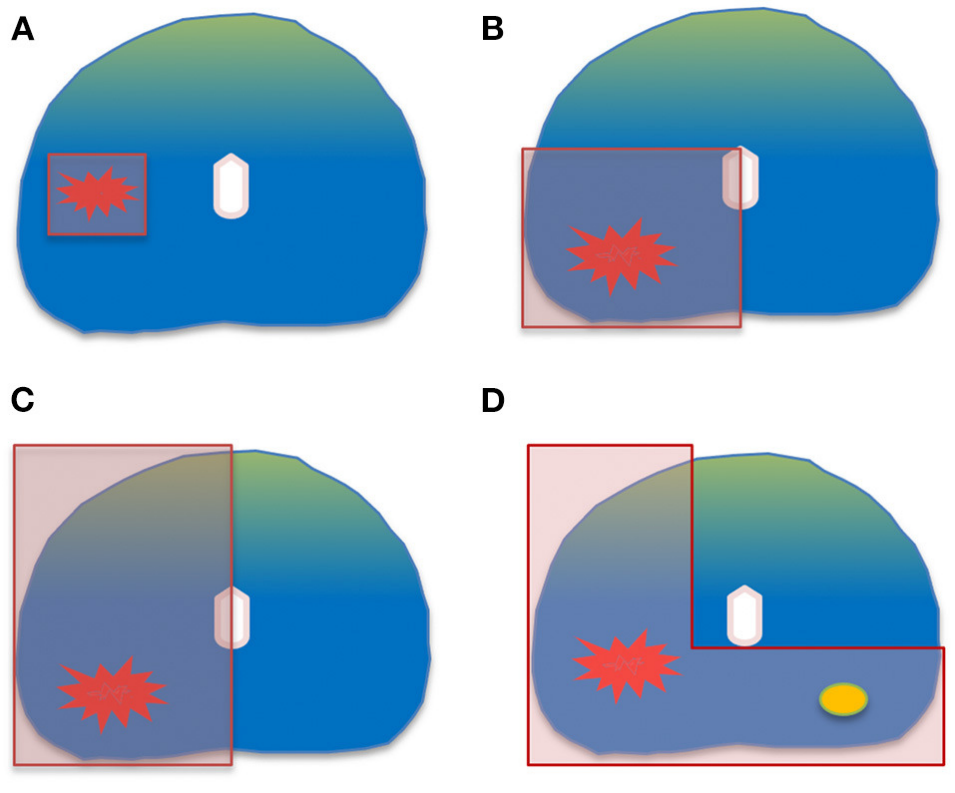
Common ablation templates: **(A)** focal ablation. **(B)** quadrant ablation. **(C)** hemi-ablation. **(D)** “hockey-stick” ablation.

The size and the location of the index lesion dictates the focal therapy strategy; coverage with a minimum of 1 cm margins around the index lesion is the priority in order to achieve local control (17–19). Treatment of a small unilateral cancer by a focal ablation will result in much less genito-urinary toxicity than treating a large portion of the gland. The following ablation templates are commonly used according to the cancer location, volume and extension on imaging and biopsy: focal ablation, zonal ablation, quadrant ablation, hemi-ablation and “hockey-stick” ablation (Figure 1) (20). The choice of the ablation template has a two-sided impact. From an oncological point of view, insufficient surgical margin is more likely to expose the patient to a higher risk of recurrence while from a functional perspective, the opposite is assumed.

Finally, different sources of energy have different side effect profiles (21). Available energies can be generally classified in thermal and non-thermal energies according to their main ablation mechanism. Among thermal sources of energy, the most used ones are: HIFU, cryotherapy, focal laser ablation and radiofrequency ablation. Among non-thermal sources of energy, the most used ones are: irreversible electroporation, PDT and brachytherapy. While it is possible to modulate for each energy the ablation template, thermal energies generally lead to a slightly wider ablation field as there is a progressive temperature gradient of thermal dispersion around the ablation target; non-thermal energy have usually a more demarcated boundary between the treated and the untreated tissue which limit the damage to the surrounding area. However, the choice of energy source should rely on patients' characteristics, intrinsic features and stage of assessment rather than on a theoretical lower side effect profile. Moreover, the field of focal therapy is rapidly evolving and novel sources of energy are constantly emerging. The potential advantages on novel technologies is yet to be confirmed in acceptable comparative studies.

### Type of Complications

Specialists performing focal therapy should be well aware of possible complications and their management. While the risk profile is more favorable than for whole gland treatments, genito-urinary toxicity and complications can occur after treatment (16). This includes peri-operative, short-term, mid-term and late complications. The types of complications and their reported frequency are summarized in Table 1 (12).

**Table 1:** Complications and their rates in the primary focal therapy setting.

**Type of complication**	**Rate**
Infectious (urinary tract infection, epididymo-orchitis)	0–17%
Haematuria	Very frequent; not reported
Acute urinary retention	0–17%
Urethral sloughing	Frequent; not reported
Urinary incontinence	0–5%
Erectile dysfunction	0–46%
Orgasmic/ejaculatory dysfunction	Not reported
Recto-urethral fistula	0–1%

#### Peri-Operative Complications

The most common complications after focal therapy usually occur within the first 30 days after the intervention (22). These are often haematuria, infectious complications or catheter related issues such as pain, discomfort and urethral sloughing. Urine culture should be routinely performed prior to the treatment to rule out an ongoing infection. A 7-day antibiotic prophylaxis is usually recommended post operatively (23). Due to the swelling of the prostate induced by focal treatment, urinary catheter is recommended for 3–10 days, depending on the treatment protocol and the cancer location. Alpha-blockers are suggested prior to trial without the catheter (TWOC) and continued for 2 weeks after treatment. Usually, catheter induced discomfort and pain will be the most frequent symptom post-operatively (24). Therefore, painkillers, anti-inflammatory and anti-muscarinic drugs should be routinely prescribed. Finally, an information leaflet explaining in detail about the possible post-operative complications should be provided for all patients. A suggested protocol after focal therapy is summarized in Table 2. This might vary according to the energy source used, the treatment template and patients' characteristics.

**Table 2:** Suggested protocol for perioperative care in focal therapy.

**Treatment**	**Duration**	**Dose**	**Frequency**
Urinary catheter	(3–10 days)^*^	x	x
Antibiotic	7 days	Depending on local guidelines	Depending on choice
Paracetamol	2 weeks	1,000 mg	PRN
Ibuprofen	2 weeks	400 mg	3 times a day
Alpha-blocker	2 weeks	Depending on treatment	Once a day
Antimuscarinic drug	Until TWOC	Depending on treatment	PRN
Information leaflet^**^	x	x	x

* *this may vary according to the energy sourced used*.

** *an information leaflet clearly explaining to the patient possible complications and actions to take after treatment*.

Urinary tract infection and epididymo-orchitis after focal therapy can occur in 0–17% of patients (12). In two recent RCTs reporting on focal PDT and HIFU peri-operative urinary infection rates were 2 and 10%, respectively (25, 26). In recently published large cohort prospective studies on focal cryotherapy and HIFU the infection rates vary between 8.5 and 9% (27, 28). The sepsis rates are poorly reported in the available literature or not separately reported. In our experience sepsis after focal treatment is very rare. A recent prospective study has reported a single shot antibiotic prophylaxis prior to HIFU treatment with similar infectious rates as in the literature (29). A consensus on antibiotic prophylaxis choice and duration for focal treatments is yet to be achieved. In our opinion, due to global rise in antibiotic resistance, each center performing focal therapy should discuss the choice of antibiotic prophylaxis with the local preventive service to match the regional resistance patterns.

About 1 in 5 men will present a temporary LUTS following focal therapy (30) as shown in focal HIFU, while acute urinary retention is reported in 0–17% (12). Patients should also be warned about possible urethral sloughing following focal therapy. The frequency and amount of debris may vary between the energy sources and the ablation template (31). In some instances, urethral sloughing and debris might block the catheter causing acute urinary retention. Most men will respond to alpha-blocker treatment with symptoms gradually disappearing in the 1st month after the intervention. Patients failing the first TWOC should keep the indwelling catheter until the post-treatment inflammation and urethral sloughing is reduced. In case of a second failed TWOC a cystoscopy under general anesthesia is advised in order to rule-out the presence of obstructive necrotic tissue that would require a transurethral resection. The need for endoscopic interventions after focal treatments has become less frequent with the advent of more conservative ablation templates (22, 27).

Less commonly reported complication is penile numbness and penoscrotal swelling. It is most common in the peri-operative period following cryotherapy and in sources of energy delivered percutaneously through the perineum; it can occur in 10% of the cases (28).

Finally, haematuria is very common after any type of focal therapy. There are no studies that report the need of a blood transfusion following the treatment, although clot retention might occasionally, especially in patients using blood thinning agents.

#### Erectile Function and Sexual Satisfaction

The impact of active treatment on patients' sexual function can be a major factor contributing to the individual choice of therapy. Two large systematic reviews reported on erectile function in men following different types of focal therapy (12, 32); overall, 54–100% of patients had erections sufficient for penetration (with or without a phosphodiesterase type 5 inhibitor). The systematic review by Walker et al. concluded that most studies assessing the outcomes of focal therapy on sexual function are not of high quality and uses heterogenous outcomes to describe erectile dysfunction. Initial results of the PART randomized control trial that assigned 82 men to either radical prostatectomy or focal ablation by HIFU are now available. Although this was a pilot trial to prove the feasibility of a larger confirmatory trial, validated PROMs confirm a clear advantage in favor of HIFU (26): the HIFU group had a significantly better outcomes concerning sexual function (OR 12.5, 95% CI 4.5–18.5) and sexual quality of life as measured by the EPIC questionnaire (OR 10.9, 95% CI 4–17.8). There was no significant difference in sexual desire between the two groups. Another RCT randomizing patients between PDT and active surveillance has shown very low (1%) erectile dysfunction rates in both groups (25). Patients receiving PDT did present a transient erectile dysfunction, however at 2 years follow-up, the mean International Index of Erectile Function 15 (IIEF-15) scores were comparable between the groups (15 for PDT and 16.8 for active surveillance; *p*-value not reported).

A combined analysis of three prospective development trials evaluating erectile dysfunction post-focal HIFU demonstrated a complete return to baseline function at 1 year. A transient erectile dysfunction was observed at 1 month with a significant decline of the IIEF-15 score (*p* < 0.01). However, at 1 year there was no significant difference in the erectile function as compared to baseline score (*p* = 0.3). The number of men requiring phosphodiesterase type 5 inhibitor treatment went to 10% pre-operatively to 37% at 1 year (14).

Ejaculatory and orgasmic dysfunction are significant side effects following active treatment of prostate cancer, although probably underestimated and underreported (33). The rates of retrograde ejaculation/anejaculation and orgasmic dysfunction following focal therapy are poorly reported in the available literature. Patients undergoing any form of focal treatment should be warned about the risk of “dry orgasm” after treatment. This is not harmful and generally does not affect sexual pleasure.

It is important to highlight that ongoing trials are underway to evaluate sexual function after focal therapy. For instance, a trial in United Kingdom (34) will recruit patients undergoing different types of focal therapy. Patients' will fill in validated PROMs to explore ejaculation, orgasm, libido/sexual desire, masculinity/virility, penile morphology, pain or discomfort, regret, shame, cancer-related stress, overall impact and partner satisfaction. This will help to council and manage expectations of prostate cancer patients undergoing focal therapy in the future.

#### Urinary Continence

Urinary incontinence is uncommon after focal therapy, regardless the source of energy used. Pad-free continence rate varies between 95 and 100%, while leak free continence is reported in 83–100% (12). In the PART randomized controlled trial, the overall urinary quality of life (OR 6.7, 95% CI 0.8–12.6), urinary function (OR 10.8, 95% CI 4.1–17.5) and urinary incontinence (OR 22.9 95% CI 13.6–32.2) were all in favor of focal HIFU when compared to radical prostatectomy (26). At 6 months no men in HIFU group reported the need to use pads as compared to around 60% in the radical prostatectomy group. In the focal PDT vs. active surveillance randomized trial the incontinence levels were also low (1%) (25). Incontinence was mostly related to urgency and usually occurred in the initial period after catheter withdrawal. Multiple prospective studies confirm low incontinence rates (12, 27, 35) with pad-free continence rates ranging between 95 and 100%. Patients presenting urinary incontinence after focal therapy rarely require more than one pad a day, thus social continence is maintained in most cases (27) as reported with focal HIFU.

The management of urinary incontinence following focal therapy should be adapted to the degree of incontinence. In most cases the recovery will be spontaneous while some men might need pelvic floor physiotherapy. We did not find any studies or case reports describing the need for artificial urinary sphincter or other invasive procedure following focal therapy for prostate cancer.

#### Rectal Toxicity

Recto-urethral fistula is a rare complication of focal therapy. An abnormal connection between the intestinal and the urinary systems is formed resulting in pneumaturia, fecaluria and urine leakage from the rectum. In the primary focal therapy setting, recto-urethral fistula is rare at 0–1% (12). Multiple RCT and prospective studies on different types of focal therapy confirm the low rates of this complication (22, 25–28). The risk is highest when treatment is performed in a salvage setting and when cancer is located in the posterior part of the prostate and extracapsular extension is present. Initial treatment is conservative in most cases with a long duration indwelling catheter. In case of conservative treatment failure, a temporary colostomy can be considered but, in most cases, a reconstructive procedure with excision of the fistulous tract, followed by closure and mobilization of an interposition graft or flap is necessary to definitively solve the problem.

#### Focal Therapy in the Salvage Setting

Focal salvage therapy after ERBT has a completely different toxicity profile than primary focal therapy: the rate of complications is significantly higher although much lower than in salvage radical prostatectomy.

A recent systematic review by Khoo et al. has summarized the complication rates for focal therapy strategies performed after ERBT treatment (36). Grade 3 toxicity adverse events were rare with all treatment modalities: recto-urethral fistula was reported in 0–5.5%, urethral stricture in 5–10% and pubic bone osteitis in 0.7–4.2%. Pad free continence rates were around 87%. Erectile function was reported in two studies and worsened from 18 to 13 points and from 15 to 13 points, respectively, as reported with IIEF−5 PROMs. The authors acknowledge significant limitations as most studies were single arm case series with a lack of standardization in patient selection, treatment protocols and outcome reporting. There are no RCTs comparing focal salvage treatment modalities to other treatments. Focal therapy in the post ERBT setting should be performed only by experienced units. Post-radiotherapy changes in the prostate and surrounding tissues make any procedure more challenging. The procedure needs to be adapted to each case, and some devices need to be adapted with specific parameters to avoid major complications.

## Conclusion

Focal therapy has become an interesting treatment strategy for localized prostate cancer. Level 1 evidence shows its favorable toxicity profile and preservation of genito-urinary function. Most complications are mild and follow the 30-day period after treatment, these can be managed with medication and do not require invasive procedures in the majority of patients. Urinary incontinence is rare, and the risk of new onset erectile dysfunction is much lower than for whole gland treatments. The toxicity profile of focal therapy in the salvage setting has been less evaluated in robust studies, although the complication rate is higher with severe complications occurring in up to one man in 10.

## Author Contributions

AR and MV generated and produced first draft of the manuscript. GM, IH, VK, AK, FZ, FP, DT, IT, RB, CK, FC, CF, and GG revised the manuscript. All authors contributed to the article and approved the submitted version.

## Conflict of Interest

The authors declare that the research was conducted in the absence of any commercial or financial relationships that could be construed as a potential conflict of interest. The handling editor declared a shared affiliation, though no other collaboration, with one of the authors FP at the time of the review.
